# MRI phenotypes of the brain are related to future stroke and mortality in patients with manifest arterial disease: The SMART-MR study

**DOI:** 10.1177/0271678X18818918

**Published:** 2018-12-14

**Authors:** Myriam G Jaarsma-Coes, Rashid Ghaznawi, Jeroen Hendrikse, Cornelis Slump, Theo D Witkamp, Yolanda van der Graaf, Mirjam I Geerlings, Jeroen de Bresser, on behalf of the Second Manifestations of ARTerial disease (SMART) Study group

**Affiliations:** 1 Department of Radiology, University Medical Center Utrecht, and Utrecht University, Utrecht, The Netherlands; 2 MIRA Institute for Biomedical Technology and Technical Medicine, University of Twente, Enschede, The Netherlands; 3 Department of Radiology, Leiden University Medical Center, Leiden, The Netherlands; 4 Julius Center for Health Sciences and Primary Care, Department of Epidemiology, University Medical Center Utrecht, and Utrecht University, Utrecht, the Netherlands

**Keywords:** Brain imaging, cluster analysis, magnetic resonance imaging, atherosclerosis, patient outcome

## Abstract

Neurodegenerative and neurovascular diseases lead to heterogeneous brain abnormalities. A combined analysis of these abnormalities by phenotypes of the brain might give a more accurate representation of the underlying aetiology. We aimed to identify different MRI phenotypes of the brain and assessed the risk of future stroke and mortality within these subgroups. In 1003 patients (59 ± 10 years) from the Second Manifestations of ARTerial disease-Magnetic Resonance (SMART-MR) study, different quantitative 1.5T brain MRI markers were used in a hierarchical clustering analysis to identify 11 distinct subgroups with a different distribution in brain MRI markers and cardiovascular risk factors, and a different risk of stroke (Cox regression: from no increased risk compared to the reference group with relatively few brain abnormalities to HR = 10.34; 95% CI 3.80↔28.12 for the multi-burden subgroup) and mortality (from no increased risk compared to the reference group to HR = 4.00; 95% CI 2.50↔6.40 for the multi-burden subgroup). In conclusion, within a group of patients with manifest arterial disease, we showed that different MRI phenotypes of the brain can be identified and that these were associated with different risks of future stroke and mortality. These MRI phenotypes can possibly classify individual patients and assess their risk of future stroke and mortality.

## Introduction

Older patients with manifest arterial disease often also have neurodegenerative and neurovascular diseases. Neurodegenerative diseases frequently lead to brain abnormalities like cerebral atrophy.^1^ Neurovascular diseases are associated with cortical infarcts, lacunes and white matter hyperintensities (WMHs).^2,3^ Although these diseases lead to heterogeneous brain abnormalities, to date, these are most commonly analysed as separate entities.^4–6^ For example, the presence of brain infarcts has been related to stroke,^5^ WMH volume has been related to stroke and mortality,^5,7^ presence of lacunes has been associated with mortality^8^ and cerebral atrophy has been linked to stroke and mortality.^7,9^ However, a combined analysis of MRI phenotypes of the brain may show a better relation with underlying aetiology and could therefore lead to a better approximation of an individual patient’s risk of future stroke or (vascular) mortality. Although the application of MRI phenotypes of the brain in patients with manifest arterial disease is novel, a comparable approach has recently been performed in patients with mild cognitive impairment^10^ and in other research fields, including asthma,^11,12^ chronic obstructive pulmonary disease^13,14^ and breast cancer.^15^

In the present study, our first aim was to identify different MRI phenotypes of the brain in middle-aged and older patients with manifest arterial disease and relate these to clinical characteristics. Our second aim was to estimate the risk of future ischaemic stroke and mortality for each of these MRI phenotypes of the brain subgroups.

## Material and methods

### SMART-MR study

In the present study, patient data from the Second Manifestations of ARTerial disease-Magnetic Resonance (SMART-MR) study were used.^16^ The SMART-MR study is a prospective cohort study at the University Medical Center Utrecht aimed to examine risk factors and consequences of brain MRI abnormalities in patients with manifest arterial disease.^16^ Patients newly referred to the University Medical Center Utrecht for treatment of manifest arterial disease (cerebrovascular disease, peripheral arterial disease, manifest coronary artery disease or an abdominal aortic aneurysm) were invited to participate between May 2001 and December 2005. In the present study, follow-up data until March 2015 are used. During a one-day visit to the medical centre, a physical examination, blood and urine samples, neuropsychological assessment, ultrasonography of the common carotid arteries and a 1.5T brain MRI scan were performed. Questionnaires were used to assess cardiovascular risk factors, medical history, medication use and demographics. The SMART-MR study was approved by the medical ethics committee of the University Medical Center Utrecht according to the guidelines of the Declaration of Helsinki of 1975 and written informed consent was obtained from all patients.

### Study sample

Of the 1309 patients included, 19 patients had no MRI, 239 patients had one or more missing MRI sequences and 48 patients had severe motion artefacts or other artefacts in their MRI scans. As a result, a total of 1003 patients were available for the present study.

### Cardiovascular risk factors

Weight and height were measured and the body mass index was calculated (kg/m^2^). Systolic and diastolic blood pressures (mmHg) were measured with a sphygmomanometer. These measurements were repeated twice, and the average between the two measurements was calculated. Glucose and lipid levels were determined from an overnight fasting blood sample. Diabetes mellitus was defined as a glucose level of ≥ 7.0 mmol/L, a history of diabetes mellitus, reported in the questionnaire or use of oral antidiabetic drugs or insulin. Hyperlipidaemia was defined as a total cholesterol level of > 5.0 mmol/L, self-reported use of lipid-lowering drugs or a low-density lipoprotein cholesterol level of > 3.2 mmol/L. Hyperhomocysteinaemia was defined as a homocysteine level of ≥ 16.2 µmol/L. Smoking (pack-years) and drinking habits (never, past and current) were assessed by questionnaires. Ultrasonography was performed to measure the intima-media thickness (IMT) in both common carotid arteries (in mm).

### Brain MRI

MR imaging of the brain was performed on a 1.5T MRI system (Gyroscan ACS-NT, Philips Medical Systems, Best, The Netherlands) using a standardized scan protocol. Transversal T1-weighted (repetition time (TR) = 235 ms; echo time (TE) = 2 ms), T1-weighted inversion recovery (TR = 2900 ms; TE = 22 ms; TI = 410 ms), T2-weighted (TR = 2200 ms; TE = 11 ms) and FLAIR (TR = 6000 ms; TE = 100 ms; TI = 2000 ms) images were acquired with a voxel size of 0.9 × 0.9 × 4.0 mm^3^ and 38 contiguous slices. Cerebral infarcts (cortical, subcortical and lacunes) were rated by a neuroradiologist according to the STRIVE criteria.^3^ The location and affected flow territory were rated for every cerebral infarct.^17^ The flow through both internal carotid arteries and the basilar artery were determined by phase contrast imaging and summed to calculate the total CBF (ml/min).^17^

### Brain MRI features

Segmentations of white matter, grey matter, peripheral cerebrospinal fluid (CSF outside the brain), lateral ventricles and WMH were obtained by a k-nearest neighbour-based automated probabilistic segmentation method, which was performed on the T1 inversion recovery and FLAIR MRI images.^18^ Cerebral infarcts were manually segmented, and WMH segmentations were manually corrected. Total brain volume was calculated by summing the volumes white matter, grey matter, WMH and cerebral infarcts. Intracranial volume (ICV) was calculated by summing all other brain volumes. Brain volume fractions (brain parenchymal fraction, white matter fraction, grey matter fraction, peripheral CSF fraction, lateral ventricular fraction and WMH fraction) were calculated by dividing the respective brain volumes by the ICV and expressing these as a percentage of ICV.

The WMH segmentations were used in a different algorithm to automatically determine periventricular or confluent WMH (distanced ≤ 3 mm from the lateral ventricles) and deep WMH (distanced > 3 mm from the lateral ventricles).^19–21^ This classification of WMH into WMH subtypes was visually checked and corrected if necessary. The classification was used to calculate different WMH shape features per lesion (surface area, convexity, surface index and curvature, volume, solidity, complexity, eccentricity and fractal dimension).^21,22^ For more details see Table 2. The mean overall WMH per shape feature was calculated for each patient.

**Table 1.** Baseline characteristics of the patients with manifest arterial disease.

	N = 1003
Age (years)	59 ± 10
Gender, men (%)	79
Cardiovascular risk factors	
BMI (kg/m^2^)	26.8 ± 3.8
Smoking (pack years)	18 (0, 50)
Alcohol intake, former (%)	26
Hypertension (%)	52
Hyperlipidaemia (%)	80
Hyperhomocysteinaemia (%)	12
Diabetes mellitus (%)	12
IMT (mm)	0.88 (0.63, 1.25)
ApoE ԑ4 (%)	34
Arterial disease location, % (n)	
Peripheral arterial disease	22.3 (224)
Cerebrovascular disease	22.7 (228)
Coronary artery disease	57.7 (579)
Abdominal aortic aneurysm	9.2 (92)

Note: Values represent means ± SD, percentages, and medians (10th, 90th percentile).

BMI: body mass index; IMT: average intima-media thickness.

Range age: 25 to 82 years. Range BMI: 15.4 to 42.9 kg/m^2^. Percentage missing: BMI: 0.1%, Smoking: 0.5%, Alcohol intake: 0.6%, Hypertension: 0.8%, Hyperlipidaemia: 1.4%, Hyperhomocysteinaemia: 0.4%, Diabetes mellitus: 1.7%, IMT: 2.0%, ApoE: 15.8%.

**Table 2.** MRI features of the 11 subgroups with different MRI phenotypes of the brain.

Subgroup (n)	1 (n = 186)	2 (n = 51)	3 (n = 160)	4 (n = 99)	5 (n = 46)	6 (n = 60)	7 (n = 135)	8 (n = 86)	9 (n = 70)	10 (n = 51)	11 (n = 55)	*p*
Group name	Limited burden	Limited burden	Limited burden	Limited burden	Cortical infarcts	Lacunar infarcts	Limited burden	Limited burden	Mainly CSVD	Multi burden	Neuro degenerative	
Volume fractions (% ICV)												
BPF	81.8 ± 1.9	80.5 ± 2.3	80.1 ± 1.7	80.3 ± 1.9	77.6 ± 2.4	77.4 ± 2.4	77.3 ± 2.1	77.9 ± 2.1	78.2 ± 1.8	75.5 ± 2.3	74.6 ± 2.8	<0.001
WM	41.5 ± 1.6	42.4 ± 1.4	44.4 ± 1.3	42.1 ± 1.5	42.3 ± 1.7	42.4 ± 2.0	42.3 ± 1.4	43.9 ± 1.4	41.1 ± 1.8	41.3 ± 2.4	41.6 ± 2.7	<0.001
CGM	40.3 ± 2.1	38.0 ± 1.9	35.7 ± 2.1	38.1 ± 2.0	33.5 ± 2.9	34.2 ± 2.3	35.0 ± 2.5	33.8 ± 2.3	36.4 ± 2.5	32.2 ± 2.9	32.5 ± 3.0	<0.001
Lateral ventricles	1.3 ± 0.5	1.8 ± 1.0	1.7 ± 0.6	2.0 ± 0.7	2.5 ± 1.0	2.5 ± 0.9	2.1 ± 0.7	1.9 ± 0.5	2.6 ± 0.9	3.7 ± 1.4	3.4 ± 0.9	<0.001
Peripheral CSF	16.8 ± 1.8	17.8 ± 2.0	18.2 ± 1.6	17.6 ± 1.8	19.9 ± 2.3	20.1 ± 2.0	20.6 ± 1.8	20.2 ± 2.0	19.2 ± 2.0	20.8 ± 2.2	22.0 ± 2.8	<0.001
WMH	0.03 (0.01,0.11)	0.01 (0.00,0.02)	0.03 (0.01,0.07)	0.10 (0.05,0.28)	0.06 (0.03,0.18)	0.14 (0.05,0.34)	0.05 (0.02,0.13)	0.12 (0.05,0.29)	0.38 (0.17,0.85)	1.13 (0.62,3.01)	0.27 (0.07,0.60)	<0.001^a^
Blood flow (ml/min)	4.2 ± 0.9	3.4 ± 0.6	3.4 ± 0.7	3.9 ± 0.7	3.2 ± 0.7	3.4 ± 0.8	3.6 ± 0.8	3.0 ± 0.6	3.2 ± 0.5	3.2 ± 1.0	3.1 ± 0.7	<0.001
WMH shape features												
Solidity	0.75 ± 0.15	0.90 ± 0.07	0.78 ± 0.11	0.36 ± 0.12	0.56 ± 0.17	0.41 ± 0.17	0.70 ± 0.16	0.31 ± 0.12	0.30 ± 0.10	0.24 ± 0.05	0.33 ± 0.10	<0.001
Convexity	1.00 ± 0.07	0.89 ± 0.05	1.01 ± 0.06	1.28 ± 0.12	1.10 ± 0.11	1.16 ± 0.12	1.02 ± 0.07	1.33 ± 0.16	1.05 ± 0.09	0.87 ± 0.13	1.13 ± 0.10	<0.001
Concavity index	1.04 ± 0.05	1.12 ± 0.05	1.02 ± 0.05	0.97 ± 0.06	1.03 ± 0.07	1.04 ± 0.07	1.04 ± 0.06	0.98 ± 0.07	1.18 ± 0.08	1.36 ± 0.10	1.11 ± 0.08	<0.001
Fractal dimension CPWMH	1.12 ± 0.16	0.85 ± 0.16	1.12 ± 0.11	1.31 ± 0.12	1.21 ± 0.13	1.33 ± 0.12	1.19 ± 0.13	1.34 ± 0.11	1.51 ± 0.11	1.68 ± 0.12	1.40 ± 0.13	<0.001
Fractal dimension DWMH	1.50 ± 0.17	1.38 ± 0.19	1.5 ± 0.27	1.45 ± 0.11	1.45 ± 0.14	1.46 ± 0.12	1.36 ± 0.14	1.48 ± 0.11	1.46 ± 0.07	1.44 ± 0.06	1.47 ± 0.12	<0.001
Eccentricity	0.45 ± 0.14	0.50 ± 0.24	0.52 ± 0.18	0.51 ± 0.13	0.50 ± 0.17	0.45 ± 0.14	0.49 ± 0.16	0.55 ± 0.13	0.44 ± 0.08	0.44 ± 0.07	0.46 ± 0.12	<0.001
DWMH, % present	45	35	44	70	78	78	61	74	68	100	84	
Infarcts, % present												
Cortical	2	16	0	0	98	38	0	2	15	29	9	<0.001
Large subcortical	0	0	0	1	0	2	1	0	6	2	0	0.031
Lacunar; WM	1	6	1	7	0	98	2	0	37	59	10	<0.001
Lacunar; Deep GM	4	2	0	7	15	18	7	2	20	55	6	<0.001

Note: Values represent means ± SD, % (OR (95% CI)) or median (10th, 90th percentile).

a Natural log transformed due to a non-normal distribution. For the between subgroup comparison, an ANCOVA was used for continues data and multinomial logistic regression was used for discrete data, both with age as a covariate and Bonferroni correction to correct for multiple testing. A *p*-value < 0.05 was considered statistically significant.

Percentage missing: Solidity/Convexity/Concavity index/FD CPWMH: 0.4%, FD DWMH/Eccentricity: 38.5%, Blood flow: 6.4%.

FD: fractal dimension; CPWMH: confluent or periventricular white matter hyperintensity; DWMH: deep white matter hyperintensity; ICV: intracranial volume; BPF: brain parenchymal fraction; WMH: white matter hyperintensity; CGM: cortical grey matter.

The post-hoc analyses using Bonferroni correction showed significant differences in the following groups: BPF: 1≠all; 2≠1,5–11; 3≠1,5–11; 4≠1,5–11; 5≠1–4,10,11; 6≠1–4,10,11; 7≠1–4,10,11; 8≠1–4,10,11; 9≠1–4,10,11; 10≠1–9; 11≠1–9; WM: 1≠2,3,7,8; 2≠1,3,8–10; 3≠1–7,9–11; 4≠3,8,9; 5≠3,8,9; 6≠1,3,8–10; 7≠1,3,8–10; 8≠1,2,4–11; 9≠2–8; 10≠2,3,6–8; 11≠3,8; CGM: 1≠all; 2≠1–3,5–11; 3≠1–6,8,10,11; 4≠1,3–11; 5≠1–4,7,9; 6≠1–4,9–11; 7≠1,2,5,8–11; 8≠1–4,7,9,10; 9≠1,2,4–11; 10≠1–4,6–9; 11≠1,4,6,7,9; lateral ventricles: 1≠all; 2≠1,5,9–11; 3≠1,5–7,9–11; 4≠1,5,6,9–11; 5≠1–4,8,10,11; 6≠1–4,7,8,10,11; 7≠1,3,6,9–11; 8≠1,5,6,9–11; 9≠1–4,7–11; 10≠1–9; 11≠1–9; Peripheral CSF: 1≠3–11; 2≠5–11; 3≠1,5–11; 4≠1,5–11; 5≠1–4,11; 6≠1–4,11; 7≠1–4,9,11; 8≠1–4,11; 9≠1–4,7,10,11; 10≠1–4,9,11; 11≠all; WMH: 1≠2,4–11; 2≠all; 3≠2–11; 4≠1–5,7,9–11; 5≠1–6,8–11; 6≠1–3,6,7,9–11; 7≠1–4,6–11; 8≠1–3,5,7–11; 9≠1–10; 10≠all; 11≠1–8,10; blood flow: 1≠1–3,5–11; 2≠1,4; 3≠1,4,8; 4≠2–6;8–11; 5≠1,4,7; 6≠1,4,8; 7≠1,5,8–11; 8≠1,3,4,6,7; 9≠1,4,7; 10≠1,4,7; 11≠1,4,7; solidity: 1≠2,4,5,8–11; 2≠all; 3≠2–11; 4≠1–3,5,7,10; 5≠all; 6≠1–3,5–11; 7≠all, 8≠1–3,5–7; 9≠1–3,5–7; 10≠1–7,11; 11≠1–3,5–7,10; convexity: 1≠2,4–6,8–11; 2≠1–9,11; 3≠2–6,8,10,11; 4≠all; 5≠1–8,10; 6≠1–10; 7≠2,4–8,10,11; 8≠all; 9≠1,2,4,6,8,10,11; 10≠1,3–11; 11≠1–4,7–11; concavity index: 1≠2,4,8–11; 2≠1–10; 3≠2–11; 4≠1–5,7,9–11; 5≠2,4,8–11; 6≠2,4,8–11; 7≠2,4,8–11; 8≠1–3,5–11; 9≠all; 10≠all; 11≠1,3–10; FD CPWMH: 1≠2,4–11; 2≠all; 3≠2–11; 4≠1–5,7,9–11; 5≠1–6,8–11; 6≠1–3,5,7,9,10; 7≠1–4,6–11; 8≠1–3,5,7,9,10; 9≠all; 10≠all; 11≠1–5,7,9,10; FD DWMH: 1≠2,4–11; 2≠all; 3≠2–11; 4≠1–5,7,9–11; 5≠1–6,8–11; 6≠1–3,5,7,9,10; 7≠1–4,6–11; 8≠1–3,5,7,9,10; 9≠all; 10≠all; 11≠1–5,7,9–11 and eccentricity: 1≠8; 3≠9; 6≠8; 8≠1,6,9,10; 9≠3,8; 10≠8;

### Outcomes

Patients received a questionnaire every six months to provide the investigators information on hospitalization and outpatient clinic visits. All possible events were audited independently by three physicians of the End Point Committee. Patients were followed until death or refusal of further participation. The primary outcomes used in this study were overall mortality, vascular-related mortality and ischaemic stroke. Vascular-related mortality was defined as death caused by a myocardial infarction, stroke, sudden death (unexpected cardiac death occurring within 1 h after onset of symptoms, or within 24 h given convincing circumstantial evidence), congestive heart failure, rupture of an abdominal aortic aneurysm or death from another vascular cause. Ischaemic stroke was defined as relevant clinical features that caused an increase in impairment of at least one grade on the modified Rankin scale, with or without a new relevant ischaemic lesion at brain imaging.^8^ Patients were followed from the date of the MRI scan until death, loss to follow-up or end of follow-up (March 2015).

### Statistical analysis

#### Identification of subgroups with different MRI phenotypes of the brain

The brain MRI features used to determine the MRI phenotypes of the brain were brain volumes (brain parenchymal fraction, white matter fraction, grey matter fraction, peripheral CSF fraction and lateral ventricular fraction), WMH features (ventricular WMH fraction per lobe, deep WMH fraction per lobe, and the shape parameters fractal dimension, solidity, convexity, concavity index and eccentricity), cerebral infarcts (number of lacunes and cortical and subcortical infarcts, cortical infarcts and number of lacunes per lobe) and cerebral blood flow (as fraction of total brain volume). These brain MRI features were normalized as *Z*-scores for normal distributed continuous variables, or otherwise scaled between 0 and 2.

**Figure 1.**
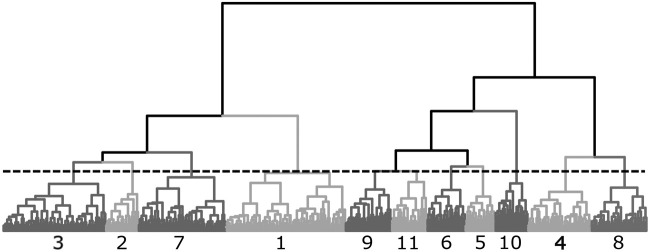
Dendrogram. The dendrogram resulting from hierarchical clustering using Ward’s criteria is visualized. The black dashed line indicates the level the dendrogram is cut to create the 11 subgroups.

**Figure 2.**
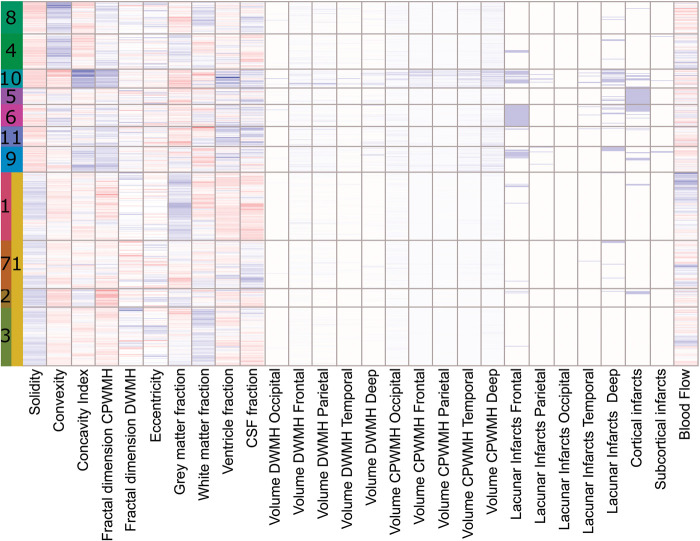
Heatmap of the hierarchical clustering results. The different colours and numbers in the first column represent the different subgroups. The subgroups are numbered based on average age (the first group is the youngest group). In the second column, the four subgroups in the bottom branch were merged resulting in the reference subgroup used for Cox regression. Each row represents one patient and each column represents a brain MRI feature used for the hierarchical clustering. Parameter values in blue are relatively high values and parameter values in red are relatively low values. For example, the Z-score of solidity for the references group is mainly above 0 and for the other groups mainly below 0. Some between-subgroup differences in brain MRI features are already visible; for example, subgroup 10 clearly has a higher concavity index, WMH volume and more cerebral atrophy, and especially subgroup 5 and 6 have a higher percentage of patients with cerebral infarcts compared to the other subgroups. CPWMH: confluent or periventricular white matter hyperintensities; DWMH: deep white matter hyperintensities; CSF: cerebrospinal fluid.

To obtain MRI phenotypes of the brain, hierarchical clustering with Ward’s criteria was performed^15^ using R version 3.3.2 and packages: NbClust,^23^ clValid^24^ and R.Matlab.^25^ Hierarchical clustering is an iterative algorithm that groups patients together based on similarities in brain MRI features. A level is a new joining of groups. Therefore, at each increasing level, the number of groups decreases. These different levels of grouping from an individual patient to one large group can be visualized using a dendrogram (Figure 1). To obtain subgroups for identification of the MRI phenotypes of the brain, the dendrogram needs to be cut at a certain level. The optimal level for this cut was determined by assessment of the average silhouette width and the Dunn index (Supplementary material Figure 3) and was also based on the heatmap (Figure 2).

Brain MRI features and cardiovascular risk factors were compared between subgroups with a different brain imaging phenotype using analysis of covariance (ANCOVA) for continuous variables and multinomial logistic regression for variables with discrete values, both corrected for age and sex. IMT and WMH were log transformed for these analyses due to a non-normal distribution. A Bonferroni correction was used to correct for multiple testing. A *p*-value of 0.05 or smaller was considered statistically significant.

#### Outcome assessment

Cox regression was used to estimate the associations between MRI phenotypes of the brain and future ischaemic stroke, mortality and vascular-related mortality, adjusted for age and sex. The reference category consisted of subgroups 1, 2, 3 and 7, as these subgroups contained relatively few brain abnormalities. We used multiple subgroups as the reference category to achieve a sufficient number of events in the reference category. These groups form the entire left branch of the dendrogram (n = 534, see Figure 1). SPSS version 21 (Chicago, IL) was used for the analyses.

## Results

A hierarchical clustering algorithm was applied on the quantified brain MRI features (brain volumes, cerebral blood flow, different types of cerebral infarcts and WMH shape features) of patients with manifest vascular disease (n = 1003). The baseline characteristics for these patients are shown in Table 1. Based on the average silhouette width, Dunn index and clustering parameters in the heatmap, the optimal cut-off was considered to be at 11 subgroups, resulting in group sizes between 46 and 188 patients (Table 2 and Supplementary Results). Subgroups were significantly different in age (*p* < 0.05) and sex (*p* < 0.05). After Bonferroni correction, differences between the age of most subgroups remained significant. See Table 2, Supplementary Table 2 and the Supplementary Results for a detailed description of between-group differences. 

### MRI phenotypes of the brain

**Figure 3.**
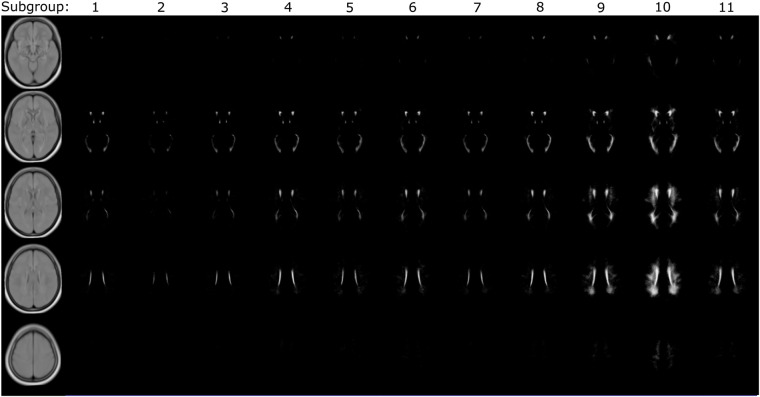
Presence of WMH per subgroup. The likelihood of WMH presence per voxel is summarized for all patients in each subgroup and visualized for five different slices. For example, patients in subgroup 10 have the most WMH lesions, where patients in subgroup 2 have the least WMH.

Brain MRI features of the 11 subgroups are shown in Table 2. Significant between-subgroup differences were found for all brain MRI features, as these were based on the hierarchical clustering classification. The following subgroups showed typical brain MRI features: subgroup 5 included patients who had mainly cortical infarcts, with presence of cortical infarcts in 98% of the study sample; in subgroup 6 mainly lacunes were found, with presence of lacunes in 98%; subgroup 9 had prominent cerebral small vessel disease (CSVD) with a relatively large WMH volume of 0.38 ml and presence of lacunes in 37% of the patients; neurodegenerative changes were mostly observed in subgroup 11 with a relatively large amount of cerebral atrophy; and a multi-burden subgroup 10 could be discerned with a relatively large amount of both cerebral atrophy and WMH volume, and presence of lacunes in 59% of the patients. The six remaining subgroups showed relatively mild brain abnormalities, characterized by few cerebral infarcts and a low CSVD burden. To illustrate the between-group differences, the probability of WMH presence is visualized per subgroup in Figure 3.

The subgroups showed significant differences with respect to age, sex, smoking, alcohol intake, hypertension, hyperhomocysteinaemia, diabetes mellitus and IMT (*p* < 0.05; see Supplementary Table 2). No between subgroup differences were found for BMI, hyperlipidaemia and number of ApoE ԑ4 carriers (*p* > 0.05).

### Outcome assessment

Of the 1003 patients, 3 patients were lost to all follow-up, 81 patients were lost to follow-up for the (vascular related) mortality and 88 patients were lost to follow-up for the ischaemic stroke outcome. For the remaining patients, the mean follow-up was 15.3 years, 217 patients had died, of whom 111 patients (51%) had vascular-related mortality, and 67 patients had a new ischaemic stroke.

**Figure 4.**
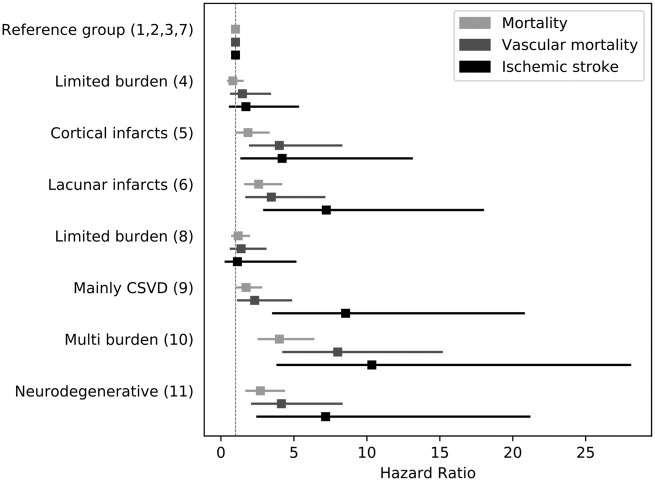
Forest plot of hazard ratios (with 95% confidence intervals) for the relationship between MRI phenotypes of the brain and outcome within the different subgroups. CSVD: cerebral small vessel disease.

The results of the Cox regression analyses (Figure 4 and Table 3) showed that, compared to the reference group with relatively few brain abnormalities (subgroups 1, 2, 3 and 7), the multi-burden subgroup had the highest increased risk of overall mortality (HR 4.00; 95% CI 2.50 to 6.40; subgroup 10), followed by the subgroup with neurodegenerative changes (HR 2.70; 95% CI 1.66 to 4.39; subgroup 11), the subgroup with mainly lacunar infarcts (HR 2.58; 95% CI 1.59 to 4.20; subgroup 6), the subgroup with mainly cortical infarcts (HR 1.85; 95% CI 1.03 to 3.34; subgroup 5), the subgroup with mainly CSVD (HR 1.72; 95% CI 1.04 to 2.83; subgroup 9). The other two groups with a limited burden (subgroups 4 and 8) showed no increased risk of overall mortality compared to the reference group with relatively few brain abnormalities.

**Table 3.** Relationship between MRI phenotypes of the brain and outcome in patients with manifest arterial disease (n=1003).

	Mortality			Vascular related mortality			Ischaemic stroke		
	No. of cases	No. per 1000 person-years	Hazard ratio	No. of cases	No. per 1000 person-years	Hazard ratio	No. of cases	No. per 1000 person-years	Hazard ratio
Reference subgroup with limited burden Subgroups 1, 2, 3, 7; n = 534	66	11.7	1 (reference)	24	4.2	1 (reference)	12	2.2	1 (reference)
Limited burden Subgroup 4; n = 99	11	10.5	0.82 (0.43–1.55)	7	6.7	1.47 (0.63–3.43)	4	3.9	1.71 (0.55–5.35)
Cortical infarcts Subgroup 5; n = 46	14	30.4	1.85 (1.03–3.34)*	11	23.9	4.00 (1.92–8.32)*	4	9.1	4.19 (1.33–13.15)*
Lacunar infarcts Subgroup 6; n = 60	23	41.2	2.58 (1.59–4.20)*	11	19.7	3.45 (1.66–7.16)*	8	15.9	7.22 (2.89–18.03)*
Limited burden Subgroup 8; n = 87	19	21.5	1.18 (0.70–1.99)	8	9.1	1.38 (0.61–3.12)	2	2.3	1.13 (0.25–5.17)
Mainly CSVD Subgroup 9; n = 71	23	34.6	1.72 (1.04–2.83)*	11	16.6	2.31 (1.10–4.88)*	11	17.9	8.54 (3.50–20.83)*
Multi burden Subgroup 10; n = 51	32	88.4	4.00 (2.50–6.40)*	23	63.5	8.00 (4.20–15.21)*	8	23.0	10.34 (3.80–28.12)*
Neurodegenerative Subgroup 11; n = 55	29	65.3	2.70 (1.66–4.39)*	16	36.1	4.14 (2.06–8.34)*	6	15.0	7.17 (2.42–21.21)*

Cox regression was used with correction for age and sex. In the table, the results are shown by giving the hazard ratio with the 95% confidence interval. Note: Differences in outcome of the different subgroups were compared to the combined reference group. *p-value < 0.05.

Vascular-related mortality is defined as: Death caused by myocardial infarction, stroke, sudden death (unexpected cardiac eath occurring within 1 h after onset of symptoms, or within 24 h given convincing circumstantial evidence), congestive heart failure, rupture of abdominal aortic aneurysm or death from another vascular cause.

Ischaemic stroke is defined as: Relevant clinical features that caused an increase in impairment of at least one grade on the modified Rankin scale, with or without a new relevant ischaemic lesion at brain imaging.

CSVD: cerebral small vessel disease.

Compared to the reference group with relatively few brain abnormalities (subgroups 1, 2, 3, and 7), the multi-burden subgroup had the highest increased risk of vascular-related mortality (HR 8.00; 95% CI 4.20 to 15.21; subgroup 10), followed by the subgroup with neurodegenerative changes (HR 4.14; 95% CI 2.06 to 8.34; subgroup 11), the subgroup with mainly cortical infarcts (HR 4.00; 95% CI 1.92 to 8.32; subgroup 5), the subgroup with mainly lacunar infarcts (HR 3.45; 95% CI 1.66 to 7.16; subgroup 6) and the mainly CSVD subgroup (HR 2.31; 95% CI 1.10 to 4.88; subgroup 9). The other two groups with a limited burden (subgroups 4 and 8) showed no increased risk of vascular mortality compared to the reference group with relatively few brain abnormalities.

Compared to the reference group with relatively few brain abnormalities (subgroups 1, 2, 3, and 7), the multi-burden subgroup had the highest increased risk of future ischaemic stroke (HR 10.34; 95% CI 3.80 to 28.12; subgroup 10), followed by the subgroup with mainly CSVD (HR 8.54; 95% CI 3.50 to 20.83; subgroup 9), the subgroup with mainly lacunar infarcts (HR 7.22; 95% CI 2.89 to 18.03; subgroup 6), the subgroup with neurodegenerative changes (HR 7.17; 95% CI 2.42 to 21.21; subgroup 11) and the subgroup with mainly cortical infarcts (HR 4.19; 95% CI 1.33 to 13.15; subgroup 5). The other two groups with a limited burden (subgroups 4 and 8) showed no increased risk of ischaemic stroke compared to the reference group with relatively few brain abnormalities.

## Discussion

In this study in middle-aged and older patients with manifest arterial disease, we identified different MRI phenotypes of the brain with hierarchical clustering of brain MRI features. We showed that these different MRI phenotypes of the brain were associated with a difference in risk of ischaemic stroke, vascular-related mortality and overall mortality.

Multiple neurodegenerative and neurovascular diseases are often present within one patient. As a single disease frequently leads to multiple brain abnormalities and brain abnormalities show overlap between diseases, it is difficult to discriminate all underlying brain diseases in one patient.^26^ Previous approaches have mainly focused on assessing single to a few brain MRI features for identification of different neurodegenerative and neurovascular diseases.^4–6^ Indeed, some diseases can be discriminated based on a single brain MRI feature or a combination of a few brain MRI features. An example of such a disease is cerebral amyloid angiopathy, which is characterized by lobar microbleeds and superficial siderosis.^27^ However, most other neurodegenerative and neurovascular diseases are often difficult to discriminate solely based on single brain MRI features. As most brain diseases lead to a specific pattern of brain abnormalities, MRI phenotypes of the brain might be used to identify previously unknown brain diseases or combinations of brain diseases by their specific pattern of brain abnormalities. This is possibly a completely new field of research.

This concept of identifying imaging phenotypes has already been performed to identify phenotypes in other types of diseases, including clinical asthma phenotypes^11,12^ and subphenotypes of COPD^13,14^ or differences in DNA methylation and gene expression in breast cancer.^15^ Two previous studies have assessed brain phenotypes.^10,28^ In one of these studies, the distribution of WMH was studied in healthy older individuals by multiple correspondence analysis.^28^ They found three distinct patterns of WMH and showed that these patterns were associated with age, hypertension and cognitive functioning.^28^ The other previous study investigated the distribution of brain MRI, cerebrospinal fluid and serum markers across individuals diagnosed with mild cognitive impairment by cluster analysis.^10^ They found four distinct patterns of markers and showed that these patterns were associated with a different risk for conversion to Alzheimer’s dementia.

To the best of our knowledge, our study is the first to identify different MRI phenotypes of the brain by assessing different vascular and non-vascular quantitative brain MRI markers in patients with manifest arterial disease. With our elaborate approach using multiple brain imaging features in patients with manifest arterial disease, we found several MRI phenotypes of the brain that were associated with a different risks of future stroke and (vascular) mortality. MRI phenotypes of the brain might in the future be used to identify individual patients that could benefit from personalized medicine approaches to prevent adverse outcome. To pave the way for clinical use, a future prediction study would be useful to confirm our results next to validation studies in other populations to confirm the external validity of our study. Furthermore, image processing software needs to be developed, tested and implemented in medical centres. This software should be fully automated and should be accurate and robust in classifying individual patients according to their MRI phenotype of the brain. Currently, a growing number of software vendors are bringing their image processing software closer to clinical practice, which helps in the future integration of software to determine MRI phenotypes of the brain.

The strengths of our study are the approach in which we combine different brain MRI features to assess MRI phenotypes of the brain and the use of this approach in a large cohort of patients with manifest arterial disease with a long follow-up duration (15 years). A strength of our technical approach includes the use of automatic brain MRI features by segmentation of brain volumes, including WMH, which also enabled us to include novel WMH shape features.^22^ Furthermore, our approach of assessing MRI phenotypes of the brain is robust, as it allows different MRI features to be used within the same method.

A limitation of our study could be the limited number of events in some subgroups, especially in the subgroups with few brain abnormalities, even with a mean follow-up of 15 years. To meet this limitation, we decided to combine subgroups with few brain abnormalities as a reference group. A potential technical limitation of our study is that patients were scanned on a 1.5 T MRI scanner that included a 2D FLAIR sequence with a slice thickness of 4 mm. This influenced the results of the WMH shape features, especially for small WMH lesions, which could have led to an underestimation of group differences. On the other hand, the influence on the more clinically relevent larger WMH was more limited. The 1.5 T MRI scanners are nowadays more and more replaced by 3 T MRI scanners, because of the potential of higher resolution images and improved visualization and sensitivity for ischaemic lesions. However, our study started with baseline MRI scans over 17 years ago when 3 T MRI was less widely available. Another technical limitation could be that, although hierarchical clustering is a machine learning method that is not biased by assumptions, some choices such as the number of subgroups need to be made that may be arbitrary. To limit this subjectivity, we used quantitative evaluation measures such as the average silhouette width and Dunn index to determine the most appropriate number of subgroups (see Suplementary materials).

In conclusion, within a group of middle-aged and older patients with manifest arterial disease, we identified subgroups with different MRI phenotypes of the brain and showed that there was a difference in risk of future stroke and mortality between these subgroups. These MRI phenotypes of the brain can possibly be used to classify individual patients and assess their risk of future stroke and mortality.

## Supplemental Material

Supplemental material for MRI phenotypes of the brain are related to future stroke and mortality in patients with manifest arterial disease: The SMART-MR study Click here for additional data file. Supplemental Material for MRI phenotypes of the brain are related to future stroke and mortality in patients with manifest arterial disease: The SMART-MR study by Myriam G Jaarsma-Coes, Rashid Ghaznawi, Jeroen Hendrikse, Cornelis Slump, Theo D Witkamp, Yolanda van der Graaf, Mirjam I Geerlings, Jeroen de Bresser and on behalf of the Second Manifestations of ARTerial disease (SMART) Study group in Journal of Cerebral Blood Flow & Metabolism
