# Supplementary material for: MRI phenotypes of the brain are related to future stroke and mortality in patients with manifest arterial disease: The SMART-MR study
Source: J Cereb Blood Flow Metab. 2018 Dec 14;40(2):354–64. doi: 10.1177/0271678X18818918 (PMC6985990; doi:10.1177/0271678X18818918)
Supplement: Supplemental material for MRI phenotypes of the brain are related to future stroke and mortality in patients with manifest arterial disease: The SMART-MR study [file Supplemental_Material11.pdf]

Supplementary Tables and figures

Supplementary Table 1. WMH shape features for the subgroup analysis.

Name	Description	Formula
Convexity (C) (1)	Describe the extent to which the shape is convex or concave. A fully convex shape has a convexity and solidity of 1. The solidity will decrease and the convexity increase as the shape becomes more concave.	
Solidity (S) (1)		
Concavity index (CI) (1)	The concavity index is a measure of roughness and can be used to differentiate between dens and irregular or elongated and curved WMH.	
Fractal dimension (FD) (2,3)	The Minkowski-Bouligand dimension (box-counting dimension) is a measure for textural roughness.	With n as the number of boxes and r the box size.
Eccentricity (E) (4,5)	Eccentricity describes the deviation from a circle. The eccentricity of a circle is one and the eccentricity of a line is zero.<br><br>The major axis denotes the largest diameter of the lesions in 3D and minor axis the smallest diameter orthogonal to the major axis.	

This table describes the definitions of the used shape features and shortly describes interpretation of the shape feature values.

Supplementary Table 2. Demographics and clinical characteristics of the eleven subgroups with different MRI phenotypes of the brain.

Subgroup (n)	1 (n=186)	2 (n=51)	4 (n=99)	5 (n=46)	6 (n=60)	7 (n=135)	8 (n=86)	9 (n=70)	10 (n=51)	11 (n=55)	P value
Group name	Limited burden	Limited burden	Limited burden	Cortical infarcts	Lacunar infarcts	Limited burden	Limited burden	Mainly CSVD	Multi burden	Neurodegenerative	
Age (years)	51±9	52±9	57±10	60±10	61±8	62±8	63±7	65±7	69±6	70±7	<0.001
Sex, % men	68%	92%	63%	80%	77%	88%	89%	83%	80%	89%	<0.001
BMI (kg/m2)	27±4	27±4	27±4	27±4	27±3	27±4	27±4	26±4	26±3	27±4	0.292
Smoking (pack years)	14 (0, 43)	17 (0, 57)	13 (0, 42)	26 (1, 72)	20 (0, 63)	24 (0, 60)	24 (0, 49)	25 (0, 53)	15 (0, 67)	13 (0, 70)	0.004
Alcohol intake, never/former	29%	14%	30%	15%	28%	20%	31%	23%	20%	42%	0.030
Hypertension	42%	51%	54%	57%	65%	50%	54%	59%	69%	67%	0.006
Hyperlipidaemia	81%	82%	75%	73%	83%	84%	83%	84%	78%	73%	0.385
Hyperhomocysteinemia	10%	2%	9%	9%	12%	11%	9%	16%	36%	37%	0.002
Diabetes mellitus	12%	8%	20%	22%	27%	27%	32%	13%	34%	38%	0.002
IMT (mm)	0.80 (0.62, 1.11)	0.87 (0.55, 1.27)	0.85 (0.63, 1.20)	0.98 (0.64, 1.38)	0.89 (0.68, 1.58)	0.92 (0.72, 1.26)	0.98 (0.65, 1.46)	0.98 (0.88, 1.35)	1.02 (0.78, 1.47)	1.00 (0.74, 1.34)	0.001*
ApoE E4	33%	52%	37%	36%	35%	37%	26%	36%	31%	28%	0.392

Values represent means ± SD, percentages, medians (10th, 90th percentile). BMI: Body mass index, IMT: average intima-media thickness, *Natural log transformed due to a non-normal distribution. For the between subgroup comparison, an ANCOVA was used for continues data and multinomial logistic regression was used for discrete data, both with age and sex as a covariate and Bonferroni correction to correct for multiple testing. A p-value < 0.05 was considered statistically significant. The post-hoc analyses using Bonferroni correction shows significant differences in the following groups: age: 1≠4-11; 2≠4-11; 3≠5-11; 4≠1,2,7-11; 5≠1-3,9-11; 6≠1-3,10,11; 7≠1-4,10,11; 8≠1-4,10,11; 9≠1-5; 10≠1-8, 11≠1-8; smoking: 1≠7 and IMT: 1≠5-11; 2≠8-11; 3≠5-11; 4≠8-11; 5≠1,3; 6≠1,3; 7≠1,3; 8≠1-4; 9≠1-4; 10≠1-4; 11≠1-4.

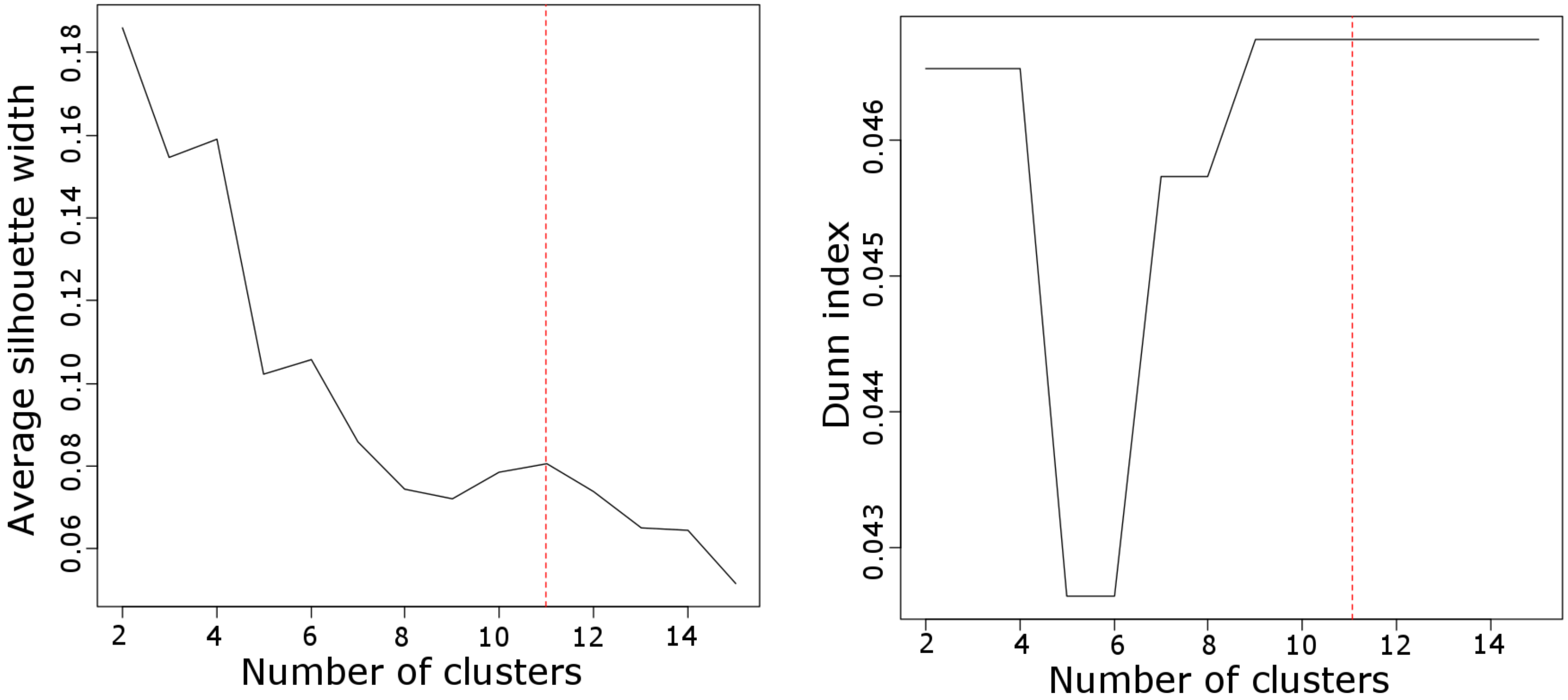

Supplementary Figure 1. Assessment of the optimal number of subgroups

On the left the average silhouette width for 2 - 15 subgroups is plotted. The silhouette width is the average of the silhouette values of all the subgroups. The silhouette value measures the degree of confidence in the clustering assignment of a particular observation. A well clustered observation has a value near 1 and poorly clustered observations have values near -1. The average silhouette width (ASW) should be maximized. Peaks are observed at 2, 4, 6 and 11 subgroups. However, the average silhouette width values will get lower with more clusters. On the right the Dunn Index for 2 - 15 subgroups is plotted. The Dunn Index is a ratio of the smallest distance between observations of different subgroups and the largest distance within the subgroup. The Dunn Index ranges between 0 and infinity and should be maximized. Between 9 and 15 subgroups, the Dunn Index is the highest.

Supplementary results

Subgroup 1 (n=186; mean age= 51±9 years) and 3 (n=160; mean age=54±8 years) have few brain abnormalities. These subgroups have a low WMH burden (0.03 ml (0.02, 0.06) and 0.03 ml (0.02, 0.05); medians (10th, 90th percentile)) with several small, solid and smooth WMH lesions around the lateral ventricles (convexity: 1.00±0.07 and 1.01±0.06; solidity: 0.75±0.15 and 0.78±0.11). These subgroups are characterized by only minor brain atrophy (BPF: 81.8±1.9 and 80.1±1.7 %) and a low number of cerebral infarcts (lacunar infarcts: 5% and 1%; cortical infarcts: 2% and 0%). The main differences between both subgroups are found in eccentricity (0.45±0.05 and 0.52±0.18), white matter fraction (41.5±1.6 and 44.4±1.3 %), cortical grey matter fraction (40.3±2.1 and 35.7±2.1 %) and blood flow (4.2±0.9 and 3.4±0.7 ml/min).

These two subgroups with relatively few brain abnormalities are relatively young (age: 51±9 and 54±8 years) with a relative low IMT (0.80 mm (0.68, 0.93) and 0.80 mm (0.67,0.97)) and have a relatively low prevalence of hypertension (42% and 41%).

Subgroup 2 (n=51; mean age=52±9 years) also has few brain abnormalities, with the lowest burden of WMH (0.01 ml (0.00, 0.01)) with only small, solid and smooth WMH lesions around the lateral ventricles (convexity: 0.89±0.05 and solidity: 0.90±0.07). This subgroup is characterized by minor brain atrophy (BPF: 80.5±2.3 %) and few cerebral infarcts (lacunar infarcts: 6% and cortical infarcts 16%).

This subgroup with relatively few brain abnormalities is relatively young (age: 52±9 years), has a cardiovascular risk factor profile comparable to subgroup 1 and 3. However, a relatively large number of patients in this subgroup consume alcohol (86%) and are ApoE ε4 carriers (52%).

Subgroup 4 (n=99; mean age= 57±10 years) has moderate brain abnormalities with an intermediate WMH burden (0.10 ml (0.06, 0.16)), consisting of PVWMH lesions that are elongated and smooth (solidity: 0.36±0.12 and convexity: 1.28±0.12). In contrast to the small, solid and smooth WMH lesions of subgroup 1, 2 and 3. This subgroup is characterized by only minor brain atrophy (BPF: 80.3±1.9 %), a low number of cerebral infarcts (lacunar infarcts 13% and cortical infarcts 0%) and relatively high cerebral blood flow (3.9±0.7 ml/min).

This subgroup has relatively few brain abnormalities, is of intermediate age (57±10 years) and has a cardiovascular risk factor profile that is comparable to subgroup 1 and 3.

Subgroup 5 (n=46; mean age= 60±10 years) is characterized by a high number of cortical infarcts (98%), a low number of lacunar infarcts (15%) and intermediate brain atrophy (BPF: 77.6±2.4 %). This subgroup has a low WMH burden (0.06 % (0.04, 0.09)) consisting of relative elongated PVWMH lesions (solidity: 0.56±0.17, convexity: 1.10±0.11).

This subgroup with predominantly cortical infarcts, is of intermediate age (60±10 years) and has a relatively large IMT (0.98 mm (0.75,1.25)).

Subgroup 6 (n=60; mean age=61±8 years) is characterized by a high number of lacunar infarcts (98%), an intermediate number of cortical infarcts (38%) and intermediate brain atrophy (BPF: 77.4±2.4 %). This subgroup has moderate brain abnormalities with an intermediate WMH burden (0.14 % (0.07, 0.23)) consisting of relative elongated WMH (solidity: 0.41±0.17) with an increased roughness of PVWMH lesions compared to subgroup 4 (convexity: 1.16±0.12).

This subgroup with predominantly lacunar infarct, is of intermediate age (61±8 years), has a relatively high number of patients with hypertension (65%) and diabetes mellitus (27%), and has an intermediate IMT thickness (0.89 mm (0.77, 1.13)).

Subgroup 7 (n=135; mean age=62±8 years) has few brain abnormalities with a low WMH burden (0.05 % (0.03, 0.08)) with relatively solid (0.70±0.16) and smooth (1.02±0.07) PVWMH lesions. This subgroup is characterized by intermediate brain atrophy (BPF: 77.3±2.1 %) and a low number of cerebral infarcts (lacunar infarcts: 9%, cortical infarcts: 0%).

This subgroup has relatively few brain abnormalities, is of intermediate age (62±8 years), has the heaviest smokers (26±22) and has a relatively high number of patients with diabetes mellitus (27%).

Subgroup 8 (n=134; mean age=63±7 years) has moderate brain abnormalities with an intermediate WMH burden (0.12 % (0.07, 0.19)) consisting of elongated and smooth PVWMH lesions (solidity: 0.31±0.12; convexity: 1.33±0.16). This subgroup is characterized by intermediate brain atrophy (BPF: 77.9±2.1 %) and a low number of cerebral infarcts (lacunar infarcts: 2%, cortical infarcts: 2%).

This subgroup has relatively few brain abnormalities, is of intermediate age (63±7 years), has a relatively large IMT (0.98 mm (0.85, 1.18) and a large number of patients with diabetes mellitus (32%).

Subgroup 9 (n=70; mean age= 65±7 years) has more severe brain abnormalities, with an intermediate WMH burden (0.38 % (0.22, 0.59)) with large PVWMH lesions with increased roughness (solidity: 0.30±0.10 and convexity: 1.05±0.09). This subgroup is characterized by intermediate brain atrophy (BPF: 78.2±1.8 %) and a high number of patients with lacunar infarcts (44%) and relatively more patients with cortical infarcts (15%).

This subgroup with predominantly features of cerebral small vessel disease, has a relatively old age (65±7 years), a large IMT (0.98 mm (0.85, 1.12)) and a large number of patients with hypertension (59%).

Subgroup 10 (n=51; mean age=69±6 years) has severe brain abnormalities with a high WMH burden (1.13 % (0.91, 2.03)) with large PVWMH lesions with increased roughness (solidity: 0.24±0.05 and convexity: 0.87±0.13). This subgroup is characterized by more severe brain atrophy (BPF: 75.5±2.3 %) and a high number of patients with cerebral infarcts (lacunar infarcts: 78% and cortical infarcts 29%).

This subgroup with predominantly multi burden, is relatively old (69±6 years), has a large IMT (1.02 mm (0.93, 1.16)), a large number of patients with hypertension (69%), hyperhomocysteinemia (36%) and diabetes mellitus (34%).

Subgroup 11 (n=55; mean age=70±7 years), has severe brain abnormalities characterized by more severe brain atrophy (BPF: 74.6±2.8 %) and a relatively low number of patients with cerebral infarcts (lacunar infarcts: 13% and cortical infarcts: 9%). This subgroup has an intermediate WMH burden (0.27 % (0.12, 0.37)) with large PVWMH lesions with only slightly increased roughness (solidity: 0.33±0.10 and convexity: 1.13±0.10).

This subgroup with predominantly neurodegenerative features, is relatively old (70±7 years), has a large IMT (1.00 mm (0.85, 1.15)), a large number of patients with hypertension (67%), hyperhomocysteinemia (37%) and diabetes mellitus (38%).

References

1. Liu EJ, Cashman K V., Rust AC. Optimising shape analysis to quantify volcanic ash morphology. GeoResJ. 2015;8:14–30.

2. Zhang L, Liu JZ, Dean D, Sahgal V, Yue GH. A three-dimensional fractal analysis method for quantifying white matter structure in human brain. J Neurosci Methods. 2006;150(2):242–53.

3. Esteban FJ, Sepulcre J, de Miras JR, Navas J, de Mendizábal NV, Goñi J, et al. Fractal dimension analysis of grey matter in multiple sclerosis. J Neurol Sci. 2009;282(1):67–71.

4. Murphy K, van Ginneken B, Schilham AMR, De Hoop BJ, Gietema HA, Prokop M. A large-scale evaluation of automatic pulmonary nodule detection in chest CT using local image features and k-nearest-neighbour classification. Med Image Anal. 2009;13(5):757–70.

5. Loizou CP, Pattichis CS, Seimenis I, Pantziaris M. Quantitative analysis of brain white matter lesions in multiple sclerosis subjects. In: Information Technology and Applications in Biomedicine, 2009 ITAB 2009 9th International Conference on. IEEE; 2009. p. 1–4.
